# Insularity effects on bird immune parameters: A comparison between island and mainland populations in West Africa

**DOI:** 10.1002/ece3.2788

**Published:** 2017-04-14

**Authors:** Elisa Lobato, Claire Doutrelant, Martim Melo, Sandra Reis, Rita Covas

**Affiliations:** ^1^ CIBIO/InBioCentro de Investigação em Biodiversidade e Recursos GenéticosUniversidade do PortoVairãoPortugal; ^2^ CEFECentre d'Ecologie Fonctionnelle et EvolutiveCNRS UMR 5175Montpellier Cedex 5France; ^3^ Percy FitzPatrick Institute of African OrnithologyDST‐NRF Center of ExcellenceUniversity of Cape TownRondeboschSouth Africa; ^4^ Departamento de BiologiaFaculdade de CiênciasUniversidade do PortoPortoPortugal

**Keywords:** Ecoimmunology, Gulf of Guinea, island adaptations, oceanic islands

## Abstract

Oceanic islands share several environmental characteristics that have been shown to drive convergent evolutionary changes in island organisms. One change that is often assumed but has seldom been examined is the evolution of weaker immune systems in island species. The reduction in species richness on islands is expected to lead to a reduced parasite pressure and, given that immune function is costly, island animals should show a reduced immune response. However, alternative hypotheses exist; for example, the slower pace of life on islands could favor the reorganization of the immune system components (innate vs. acquired immunity) on islands. Thus far, few island species have been studied and no general patterns have emerged. Here, we compared two immune parameters of birds from São Tomé and Príncipe islands to those of their close relatives at similar latitudes on the mainland (Gabon, West Africa). On islands, the acquired humoral component (total immunoglobulins) was lower for most species, whereas no clear pattern was detected for the innate component (haptoglobin levels). These different responses did not seem to arise from a reorganization of the two immune components, as both total immunoglobulins and haptoglobin levels were positively associated. This work adds to the few empirical studies conducted so far which suggest that changes in immune parameters in response to insularity are not as straightforward as initially thought.

## 1 Introduction

Island ecosystems are in many ways simplified when compared to the mainland. Oceanic islands, in particular, are characterized by impoverished biotas and hence by a reduced diversity of predators, competitors and pathogens and by simplified ecological interactions (MacArthur & Wilson, 1967; Whittaker & Fernández‐Palacios, 2007). This is thought to lead to convergent patterns of adaptation (Blondel, 2000; Losos & Ricklefs, 2009; Whittaker & Fernández‐Palacios, 2007), such as the repeated evolution of flightlessness (Grant, 1998), shifts toward a slower life‐history strategy (Covas, 2012; Novosolov, Raia, & Meiri, 2013), or changes in secondary sexual signals (Doutrelant et al., 2016; Morinay, Cardoso, Doutrelant, & Covas, 2013) in many island animals.

A change in immune function is often also assumed as part of the convergent phenotype of insular animals (Matson & Beadell, 2010; Wikelski, Foufopoulos, Vargas, & Snell, 2004). Such changes are assumed to result from changes in parasite pressure on islands. First, islands are expected to be parasite poor as a result of the overall decrease in species richness and of founder effects during colonization (the enemy‐release hypothesis; MacArthur & Wilson, 1967; Torchin, Lafferty, & Kuris, 2001; Keane & Crawley, 2002). Although more field‐based research is necessary (Matson & Beadell, 2010), several studies did support a decrease in parasite diversity on islands. For example, some birds inhabiting remote islands (Hawaii, French Polynesia, Bermuda, and Galápagos) appear to lack the majority of avian Hemosporidian lineages, a widespread group of blood parasites (Beadell, Atkins, Cashion, Jonker, & Fleischer, 2007; Beadell et al., 2006). Reduced Hemosporidian parasite diversity was also found in the Gulf of Guinea islands (C. Loiseau, M. Melo, E. Lobato, J.S. Beadell, R.C. Fleischer, S. Reis, C. Doutrelant, R. Covas, unpublished data). Other examples include a decrease in helminth parasite richness in rodents from the Mediterranean islands (de Bellocq, Morand, & Feliu, 2002), lizards in Northern Lesser Antilles (Dobson, Pacala, Roughgarden, Carper, & Harris, 1992), or in pine martens *Martes martes* in the Baleares (Segovia et al., 2007). Second, islands are expected to show different parasite prevalence. This could mainly arise from changes in hosts’ densities that affect parasite transmission (McCallum, Barlow, & Hone, 2001). So far, however, studies have obtained mixed results showing both reduced (Pérez‐Rodríguez, Ramírez, Richardson, & Pérez‐Tris, 2013; (C. Loiseau, M. Melo, E. Lobato, J.S. Beadell, R.C. Fleischer, S. Reis, C. Doutrelant, R. Covas, unpublished data)) or increased prevalence on islands (Illera, Fernández‐Álvarez, Hernández‐Flores, & Foronda, 2015). Finally, island biogeography theory also predicts an expansion of the ecological niche of successful colonizers (MacArthur & Wilson, 1967). This would translate in insular parasite community dominated by generalists, and some recent studies supported this prediction in bird parasites from the Macaronesian and the Gulf of Guinea islands (Pérez‐Rodríguez et al., 2013; (C. Loiseau, M. Melo, E. Lobato, J.S. Beadell, R.C. Fleischer, S. Reis, C. Doutrelant, R. Covas, unpublished data)). More generalist island parasites are expected to show reduced virulence (Garamszegi, 2006). A reduction in parasite virulence in small or distant islands was also predicted by a mathematical model (Hochberg & Møller, 2001), although empirical evidence is still needed.

A reduction in parasite pressure in terms of richness, infection risk, or virulence is thought to lead to decreased parasite resistance in island hosts, given the costs of maintaining a well‐performing immune function (Lindström, Foufopoulos, Parn, & Wikelski, 2004; Wikelski et al., 2004). Furthermore, the loss of genetic variation associated with island colonization (Frankham, 1997) may entail the loss of specific resistance alleles, further leading to reduced parasite resistance and immunity (Hale & Briskie, 2007; Hawley, Sydenstricker, Kollias, & Dhondt, 2005; Ilmonen et al., 2008; Reid, Arcese, & Keller, 2003; Whiteman, Matson, Bollmer, & Parker, 2006).

However, there are alternative hypotheses; for example, the slower pace of life on islands could favor the reorganization of the immune system components on islands (Lee, 2006), and hence, the responses to insularity could be more complex than assumed. The immune system is composed of different branches that can be broadly grouped in the two distinct axes of innate and acquired immunity (but see Janeway, Travers, Walport, & Shlomchik, 2001 for a more complex view), each with different induced and constitutive elements, and comprising a majority of nonspecific and specific components, respectively (Lee, 2006; Schmid‐Hempel & Ebert, 2003). The response to parasite changes may be complex because relative costs of these different immune components are variable (Lee, 2006) and fitness benefits are not obvious due to the risk of autoimmune disease in overactivated responses (Graham, 2013; Graham et al., 2011). Hence, immune response should change with the relative benefits of immune activation, which are expected to vary under different pathogen pressures (Viney, Riley, & Buchanan, 2005).

There are well‐documented cases of exotic diseases causing extinctions on islands, such as the extinction of several Hawaiian honeycreepers following the introduction of avian malaria and a competent vector to the islands (van Riper, van Riper, Goff, & Laird, 1986; Warner, 1968; Wikelski et al., 2004). This is thought to have arisen from the inability of naïve hosts to fight foreign pathogens (reviewed in LaPointe, Atkinson, & Samuel, 2012; Wikelski et al., 2004). From a conservation perspective, it is therefore important to determine whether a change in the immune system is part of the “island syndrome,” because low levels of immune defenses in combination with other demographic factors related with island life may affect vulnerability to introduced pathogens. Furthermore, from an evolutionary perspective, understanding how immunity may or not change on islands is central to understand the evolution of other associated traits. Immune response has been suggested to be involved in life‐history trade‐offs because of the common resources shared by different functions or activities (Downs, Adelman, & Demas, 2014; Graham, 2013; Lochmiller & Deerenberg, 2000; Norris & Evans, 2000; Ricklefs & Wikelski, 2002; Sheldon & Verhulst, 1996; Zuk & Stoehr, 2002). For example, reducing investment in the immune response may allow island animals to increase resource allocation for other costly fitness related traits such as reproductive investment (Bonneaud et al., 2003) or other physiological processes (Hasselquist and Nilsson 2012). In addition, immunity is supposed to be reflected in secondary sexual characters that signal individuals’ genetic parasite resistance (Hamilton & Zuk, 1982). Lower parasite levels on islands may decrease the need to signal immune function and could therefore drive the reduction in sexual ornamentation on island species (Doutrelant et al., 2016).

Whether or not island animals have reduced immune function remains to be established as the few studies conducted so far have produced diverse results (e.g., Beadell et al., 2007; Matson, 2006; Matson & Beadell, 2010; Tompkins, Mitchell, & Bryant, 2006). Additionally, most of the hypotheses about the effects of insularity on immune function arise from the study of a few endemic species with a long evolutionary history on isolated environments, such as the case of the endemic Hawaiian avifauna. Less remote archipelagos have been subject to more colonization events, have more nonendemic species, and should therefore hold more complex parasite communities (Beadell et al., 2006; Fallon, Bermingham, & Ricklefs, 2005), which may counterbalance possible trends toward reduced immune function associated with island life.

Measuring baseline immune parameters constitutes a first step in the study of immune variation that may provide indication of immune changes on islands. In this study, we aim to add to the currently incipient knowledge on the immunity of island hosts by obtaining field measures of two parameters corresponding to different arms of the immune system in a set of island endemic birds and their close mainland relatives. We quantified total antibodies (total immunoglobulins Y) as a measure of acquired humoral component and haptoglobin levels as a measure of innate immune component. The humoral response is an important component of the acquired immune system, which includes the specific antibody response that eliminates pathogens by multiple pathways and includes memory mechanisms that reduce the costs of multiple encounters with the same parasite (Janeway et al., 2001). Circulating antibodies likely reflects the individual's genotype and past and current antigenic experiences (Nussey et al., 2014). A measure of total antibodies in a given point in time may thus reflect patterns of exposure to infection or a higher investment in protective humoral response indicative of immune strength. It may also include both causes of variation (Apanius & Nisbet, 2006; Morales et al., 2004). Haptoglobin is an acute‐phase protein with anti‐inflammatory action that is part of innate nonspecific immunity. Haptoglobin binds free hemoglobin released into circulation when the membrane of erythrocytes is destabilized under certain pathological conditions such as infection, mechanical trauma, or some genetic disorders (Dhaliwal, Cornett, & Tierney, 2004). Free hemoglobin can catalyze the formation of hydroxyl radicals and may therefore cause oxidative damage. By binding to it with high affinity, haptoglobin inhibits the oxidative activity of hemoglobin and reduces the loss of hemoglobin in urine and concomitant loss of iron (Bicho, da Silva, Medeiros, & Bicho, 2013). Although the functional meaning of baseline haptoglobin levels is not entirely clear, basal levels have been shown to be repeatable within individuals and to correlate with haptoglobin elevation under inflammatory conditions (i.e., after an endotoxin challenge) in some species (Matson, Horrocks, Versteegh, & Tieleman, 2012) but not in others (Hegemann, Matson, Versteegh, Villegas, & Tieleman, 2013). Overall, while these two measures cannot capture the complexity of the immune system, they provide an indication of whether immunological parameters differ between islands and mainland areas, therefore contributing to our understanding of how insularity affects immunity.

We conducted a comparison between island and mainland birds based on both endemic and nonendemic species pairs from the islands of São Tomé and Príncipe (Gulf of Guinea, West Africa) and their close mainland relatives in Gabon. These islands lay ca. 200 km from the nearest mainland (Gabon), but this moderate distance from the mainland has allowed the evolution of a rich endemic avifauna—in relation to their area, the highest of any island system in the world—which co‐occurs with more recent, nonendemic, colonizers and human‐mediated introduced species (Jones & Tye, 2006; Melo, 2007). This makes these islands critical areas for conservation worldwide: São Tomé Island has the 11th most irreplaceable protected area in the world (Le Saout et al., 2013), and the forests of São Tomé and Príncipe are considered the third most important in the world from a bird conservation perspective (Buchanan, Donald, & Butchart, 2011).

These islands hold an impoverished Haemosporidian parasite community both in terms of reduced diversity and prevalence (C. Loiseau, M. Melo, E. Lobato, J.S. Beadell, R.C. Fleischer, S. Reis, C. Doutrelant, R. Covas, unpublished data), suggesting they make a good model to study immune changes to the island environment. We collected field data for 11 species groupings (pairs) allowing us to conduct comparisons between a total of 21 species.

## 2 Methods

**Figure 1:**
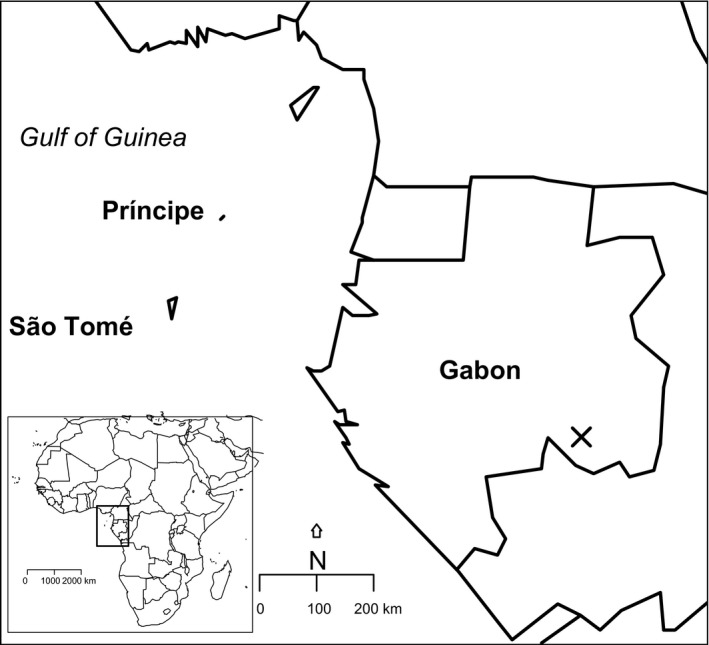
The Gulf of Guinea in Africa (inset) with the location of São Tomé and Príncipe Islands and Gabon (X: sampling site in Gabon)

The Gulf of Guinea islands form an important center of bird speciation in West Africa with 33 endemic bird species (Melo, 2007). These islands have been subject to the colonization by different bird groups that have differentiated into distinct insular species (Melo, 2007; Melo et al., 2010). The Gulf of Guinea comprises three oceanic islands (Annobón, São Tomé, Príncipe) and one land‐bridge island (Bioko), all part of the Cameroon line of volcanoes (Figure 1). In this study, we focused on the two central islands, São Tomé and Príncipe, which make the Democratic Republic of São Tomé and Príncipe, and where endemism is concentrated (28 species).

São Tomé (0^°^25′N–0^°^01′S, 6^°^28′E–6^°^45′E), with a total area of 857 km^2^, is an oceanic island in seas over 1,800 m deep. It lies 255 km W of Gabon and 150 km SSW of Príncipe Island. The island's age is estimated at over 13 Ma (Lee, Halliday, Fitton, & Poli, 1994). Similarly, Príncipe (1^°^32′N–1^°^43′N, 7^°^20′E–7^°^28′E) is an oceanic island in seas over 1,800 m deep that lies 210 km SSW of Bioko and 220 km W of the African mainland and has a total area of 139 km^2^. Príncipe Island age is estimated at 31 Ma (Lee et al., 1994).

Before their relatively recent colonization by humans, in the 16th Century, these oceanic islands were predominantly covered by moist tropical forest, and thus, the large majority of indigenous avifauna are forest birds that remain strongly associated with indigenous or secondary forest habitats or forested shade plantations (Jones & Tye, 2006). By contrast, nonendemic species are all relatively recent colonizers or species introduced in the late 19th and early 20th centuries that remain restricted to human‐altered habitats and do not occur in forest areas (Jones & Tye, 2006).

On São Tomé Island, fieldwork was carried out in February and the first week of March 2013 and January 2014 in secondary forest, abandoned shade forest plantations, and savannah. On Príncipe Island, sampling was performed in January 2014 in secondary forest, abandoned shade forest plantations, and mixed plantations. Field sampling in Gabon was performed in January 2013 around the Lekedi Park, located close to Bakoumba, approximately 70 Km SW of Franceville, in secondary forest, mixed plantations, and gardens (Figure 1). All areas sampled were at comparable altitude (below 600 m).

We sampled resident bird species that could be captured in sufficient numbers to perform paired island–mainland comparisons between conspecific populations or between closely related species (sister species whenever possible).

**Figure 2:**
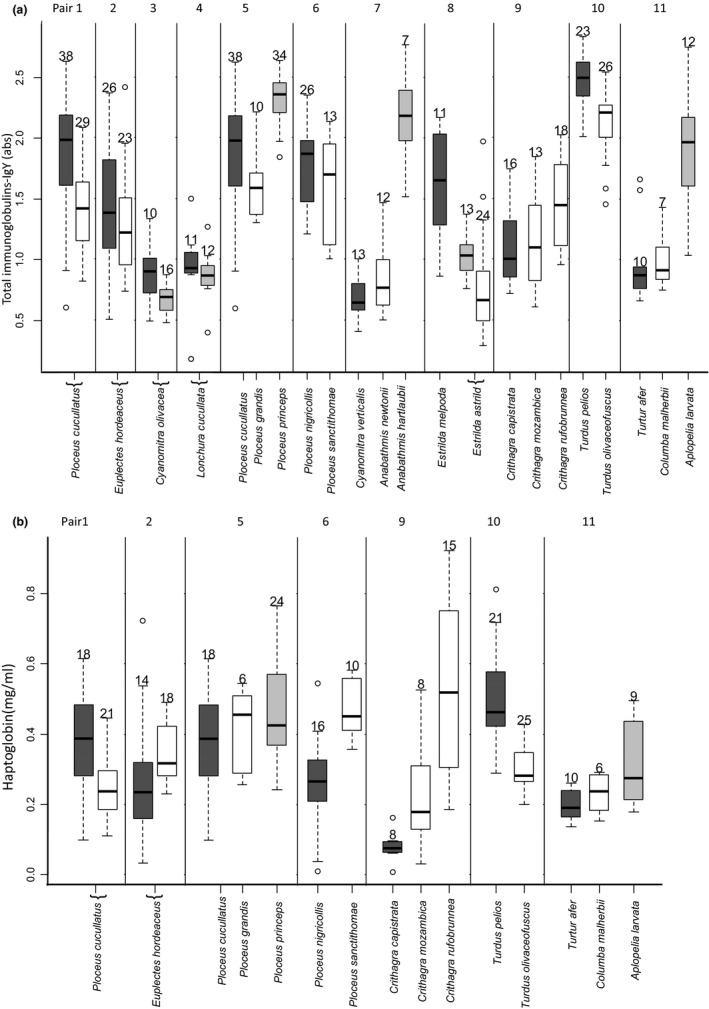
Immune parameters of paired bird species from the mainland (Gabon, dark gray) and oceanic islands (São Tomé – white; Príncipe – light gray). (a) Total immunoglobulins (abs = absorbance), a parameter of the acquired humoral immune response; (b) Haptoglobin concentration, a component of innate immunity. No haptoglobin measures are available for species from the families Nectariniidae and Estrildidae as not enough plasma volume was obtained for the majority of individuals of these families. Ploceus cucullatus from the mainland was used in two paired comparisons (see text). Box plots show median, first (Q1) and third (Q3) quartiles, upper whisker shows the maximum value lower or equal to Q3 + 1.5 × IQR (interquartile range) and lower whisker shows the minimum value higher or equal to Q1 − 1.5 × IQR. Numbers above whiskers represent sample size.

We sampled a total of 21 species from six families (*Columbidae*,* Turdidae, Nectariniidae, Ploceidae, Estrildidae, Fringillidae,* Table 1. Figure 2). Species present in both island and mainland locations were paired together (*Ploceus cucullatus, Euplectes hordeaceus, Lonchura cucullata,* and *Cyanomitra olivacea*). Otherwise we paired species according to their phylogenetic proximity (Table 1). *Ploceus cucullatus* is the closest mainland relative of both the São Tomé endemic *P. grandis* and the Príncipe endemic *P. princeps* (Staffan Andersson, Göteborg University, pers. comm.) and hence, in addition to being paired to the undifferentiated São Tomé population, it was compared to these two different species in another paired comparison (Table 1).

**Table 1:** Bird species pairs used to compare the immune parameters between islands and mainland in the Gulf of Guinea. Island(s) where species occur (“endemic status”) are in brackets: São Tomé (ST), Príncipe (P), or both locations (STP). *Ploceus cucullatus* from the mainland was used in two paired comparisons (pair 1 and pair 5, see text). N_IgY and N_hapto show sample sizes for total immunoglobulins and haptoglobin parameters

Pair	Family	Location	Species	Endemic status	N_IgY	N_hapto
Same species pairs						
1	*Ploceidae*	Gabon	*Ploceus cucullatus*	Nonendemic	38	18
		São Tomé	*Ploceus cucullatus*		29	21
2	*Ploceidae*	Gabon	*Euplectes hordeaceus*	Nonendemic	26	14
		São Tomé	*Euplectes hordeaceus*		23	18
3	*Nectariniidae*	Gabon	*Cyanomitra olivacea*	Nonendemic	10	
		Príncipe	*Cyanomitra olivacea*		16	
4	*Estrildidae*	Gabon	*Lonchura cucullata*	Nonendemic	11	
		Príncipe	*Lonchura cucullata*		12	
Different species pairs						
5	*Ploceidae*	Gabon	*Ploceus cucullatus*	Nonendemic	38	18
		Príncipe	*Ploceus princeps*	Endemic (P)	34	24
		São Tomé	*Ploceus grandis*	Endemic (ST)	10	6
6	*Ploceidae*	Gabon	*Ploceus nigricollis*	Nonendemic	26	16
		São Tomé	*Ploceus sanctithomae*	Endemic (ST)	13	10
7	*Nectariniidae*	Gabon	*Cyanomitra verticalis*	Nonendemic	13	
		Príncipe	*Anabathmis hartlaubii*	Endemic (P)	7	
		São Tomé	*Anabathmis newtonii*	Endemic (ST)	12	
8	*Estrildidae*	Gabon	*Estrilda melpoda*	Nonendemic	11	
		Príncipe	*Estrilda astrild*	Nonendemic	13	
		São Tomé	*Estrilda astrild*	Nonendemic	24	
9	*Fringillidae*	Gabon	*Crithagra capistrata*	Nonendemic	16	8
		São Tomé	*Crithagra mozambica*	Nonendemic	13	8
		São Tomé	*Crithagra rufobrunnea*	Endemic (STP)	18	15
10	*Turdidae*	Gabon	*Turdus pelios*	Nonendemic	23	21
		São Tomé	*Turdus olivaceofuscus*	Endemic (ST)	26	25
11	*Columbidae*	Gabon	*Turtur afer*	Nonendemic	10	10
		Príncipe	*Aplopelia larvata principalis*	Endemic (P)	12	9
		São Tomé	*Columba malherbii*	Endemic (STP)	7	6

Birds were captured using mist nets. Individuals were measured and weighed, and a small amount of blood was collected from the brachial vein using heparinized capillary tubes. Blood was stored in portable coolers and, at the end of the day, was centrifuged to separate the plasma from the blood cells. All plasma samples were stored at – 20°C up to the laboratory analyses. The blood cells were stored in ethanol and were used to sex monomorphic species using a molecular protocol (Griffiths, Double, Orr, & Dawson, 1998).

### 2.1 Total antibodies

We estimated total immunoglobulins (IgY), the predominant circulating antibodies in birds (Davison, Kaspers, Schat, & Kaiser, 2011) as a proxy of the acquired humoral component of the birds’ immune system. The level of these circulating antibodies was measured in plasma samples by an enzyme‐linked immunosorbent assay method using commercial anti‐chicken antibodies as described in Martínez, Tomás, Merino, Arriero, and Moreno (2003). Anti‐chicken antibodies have been previously used in antibody quantification of different bird species from diverse avian families (Cray & Villar, 2008; Lobato et al., 2011; Martínez et al., 2003; Pihlaja, Siitari, & Alatalo, 2006; Staszewski, McCoy, & Boulinier, 2008). Plasma samples were diluted 1/9,000 in carbonate–bicarbonate buffer, and 100 μl of this dilution was incubated in duplicate in ELISA plates (NUNC maxisorp, 439454) for 1 hr at 37°C and then overnight at 4°C. After washing once with phosphate‐buffered saline (PBS Tween) solution, wells were blocked with 100 μl of 5% defatted dry milk (Regilait, France) in PBS Tween during 1 hr at 37°C, and then washed with PBS Tween. Then, we added 100 μl of anti‐chicken IgY antibody conjugated with peroxidase (Sigma, A‐9046) diluted 1/1,000 in 5% defatted dry milk in PBS Tween and incubated the plates two hours at 37°C. After washing three times with PBS Tween, 100 μl of peroxidase substrate (o‐phenylenediamine dihydrochloride, SIGMA*FAST* ^™^ OPD, P9187, Sigma) was added and incubated in the dark for 15 min at room temperature. The colorimetric reaction was stopped using 50 μl of hydrochloric acid (HCl 3N). The absorbance of the resulting solution was measured at 490 nm. This absorbance provided us with a relative measure of total antibody concentration in the plasma samples, and the mean absorbance from replicated values per sample was used in the analysis. Average intra‐ and interplate coefficients of variation were 3.4% and 10.0%, respectively. To account for interplate variation, we adjusted all values to be comparable with a reference plate, running 6–16 samples in common on each plate and on the reference plate and calculating a correction equation for each corresponding plate.

Differential interspecific affinity of the anti‐chicken antibody used in the assay may hinder the comparison of total immunoglobulin levels between different bird species. To minimize this possibility, we compared either populations of the same species occurring both on the islands and on the mainland or closely related species (sister species whenever possible). Differences in protein structure between the pairs used in this study should therefore be small. To further control this issue, statistical analyses included a random factor consisting of “species” nested in “pair” nested in “family” to account for phylogenetic proximity.

### 2.2 Haptoglobin

We used a commercially available kit based on haptoglobin functional activity to determine the concentration of haptoglobin equivalent in bird plasma samples (TP801; Tri‐Delta Diagnostics). This colorimetric assay measures heme‐binding activity in the plasma and measures haptoglobin functional equivalent in birds (identified as PIT54 in chickens; Wicher & Fries, 2006). Plasma haptoglobin as measured by this functional assay can be easily quantified in a wide variety of avian species and is often used in comparative ecological studies (Matson, 2006; Millet, Bennett, Lee, Hau, & Klasing, 2007). We followed the manufacturer's instructions using 7.5 μl of plasma and measured the absorbance at 630 nm after the colorimetric reaction. Intra‐ and interplate coefficients of variation of 630‐nm absorbance averaged 3.8% and 7.8%, respectively. A standard absorbance‐known haptoglobin concentration curve was included on every run, and haptoglobin of each sample was calculated according to its corresponding curve. In addition to these measures, we scored by eye heavily hemolysed samples. When analyzing haptoglobin levels, heavily hemolysed samples were removed from the analyses, as recommended by the kit manufacturer, as these samples can strongly interfere with final results (hemolyzed samples would show higher levels of free hemoglobin, and this would lead to higher measured haptoglobin). Prescan absorbance measures prior to the addition of the final reagent can be used to account for plasma redness as suggested by Matson et al. (2012). Then, for the remaining samples, we measured a prescan absorbance value at 405 nm, which gives a quantitative measure of plasma redness‐yellowness and which gave a very strong correlation with final haptoglobin levels. We also tested the correlation of prescan levels at 450 nm for a subsample, because this measure of plasma redness has been previously used in other studies (Matson et al., 2012). Both prescan absorbance at 405 nm and 450 nm strongly correlated with our score of heavy hemolysis. However, as 405 nm explained better final haptoglobin levels in our dataset, we decided to use prescan absorbance at 405 nm as a measure of plasma coloration in our analyses.

### 2.3 Statistics

We analyzed the data with generalized linear mixed models (GLMMs) using the package nlme in R version 3.1.3 (R Core Team 2015). To account for phylogenetic dependency and for the paired structure of the analyses, we included a random term consisting of “species” nested in “pair” nested in “family” as it is a simple method to test for an effect of insularity with incomplete phylogenetic information (Covas, 2012; Doutrelant et al., 2016; Morinay et al., 2013). Nonsignificant interactions were removed when the *p*‐value of the comparison between the full model and the model without each particular interaction was >.05. *P* values for interactions cited in the text are from these model comparisons.

When analyzing the levels of circulating antibodies (immunoglobulins IgY) as the dependent variable, the explanatory variables were “insularity,” “sex,” “body mass,” “endemic status” (endemic, nonendemic), and “pair composition” (same or different species). We also tested the two‐way interactions between sex and insularity and pair composition and insularity. To further understand whether the overall insularity pattern in antibodies is mainly driven by same species pairs (see 3), we also analyzed separately the two subsets of pairs composed of the same or different species. When analyzing pairs with same species composition, we included in addition to the random factor, the explanatory variables “insularity,” “sex,” and “body mass.” When analyzing pairs with different species composition, the explanatory variables were “insularity,” “sex,” “body mass,” and “endemic status” (endemic–nonendemic).

When analyzing haptoglobin levels as the dependent variable, we included the same variables as above and added the prescan absorbance at 405 nm (“plasma redness”). The sample size used in the haptoglobin analyses is lower because plasma obtained for most small species was not enough for both antibody and haptoglobin analyses. Hence, species from the families Estrildidae (mean weight and SD = 7.9 ± 1.0 g) and Nectariniidae (10.6 ± 2.3 g) could not be included in the haptoglobin analysis.

In the subset of species excluding Estrildidae and Nectariniidae, we investigated whether the two immune parameters, antibody and haptoglobin levels, were associated. In this analysis “haptoglobin” was included as dependent variable, and as explanatory variables, we included “body mass,” “total antibodies,” “sex,” “plasma redness” “endemic status,” and “pair composition.” We also included the double and triple interactions “pair composition: antibodies:insularity” and “antibodies:sex:insularity” to investigate whether haptoglobin was expressed as a compensation of reduced antibodies in island systems, in contrast to the mainland, as suggested by Matson (2006).

To further understand whether the significant interaction “antibodies:insularity” was mainly driven by same species pairs (see 3), we also analyzed separately the two subsets of pairs composed of the same or different species. In pairs composed by populations from the same species, we used the variables “body mass,” “total antibodies,” “sex,” “plasma redness,” and the double and triple interactions “antibodies:sex:insularity”; in pairs composed of different species, we also included the variable “endemic status”.

## 3 Results

### 3.1 Antibody levels

Mainland species showed significantly higher levels of circulating antibodies than island birds (Table 2; Figure 2a). No effect of mass, sex, endemic status, or pair composition was found (Table 2). The interaction between sex and insularity was not significant (χ^2^ = 3.21, *p* = .073), and neither was the interaction of pair composition and insularity (χ^2^ = 0.050, *p* = .82). When analyzing the two subsets of pair composition separately (same species, different species), in the subset including pairs with same species composition, antibodies were significantly and positively associated with body mass (*p* < .001) and also significantly associated with insularity (*p* < .001), but in the subset with pairs with different composition, no significant effects were found (insularity, *p* = .28).

**Table 2:** Immune parameters in relation to insularity in birds from the Gulf of Guinea. Estimates correspond to average slopes for continuous explanatory variables and average differences between levels of the fixed factors. Statistics of variables are shown in bold when *p*‐value <.05

	Value estimate	*SE*	*t*‐value	*p*‐value
Total immunoglobulins *N* = 491				
(Intercept)	1.52	0.23	6.57	<.0001
Insularity (mainland)	**0.28**	**0.056**	**4.95**	**<.0001**
Body mass	0.0025	0.0022	1.12	.26
Sex (female)	−0.062	0.034	−1.80	.072
Status (nonendemic)	−0.40	0.21	−1.93	.082
Pair composition (same)	−0.19	0.24	−0.79	.47
Haptoglobin *N* = 247				
(Intercept)	0.009	0.049	0.18	.86
Plasma redness	**1.032**	**0.062**	**16.64**	**<.0001**
Insularity (mainland)	0.018	0.021	0.86	.39
Body mass	−0.00018	0.0004	−0.45	.65
Sex (female)	−0.018	0.013	−1.43	.15
Status (nonendemic)	−0.091	0.048	−1.92	.096
Pair composition (same)	0.029	0.057	0.51	.66

### 3.2 Haptoglobin levels

There was no significant effect of insularity (Table 2, Figure 2B). The interactions between sex and insularity and pair composition and insularity were not significant (χ^2^ = 2.43, *p* = .12, χ^2^ = 0.15, *p* = .70, respectively). Haptoglobin concentration was positively associated with plasma redness. There was no significant effect of mass, sex, endemic status, or pair composition (Table 2).

### 3.3 Association haptoglobin–antibodies

**Figure 3:**
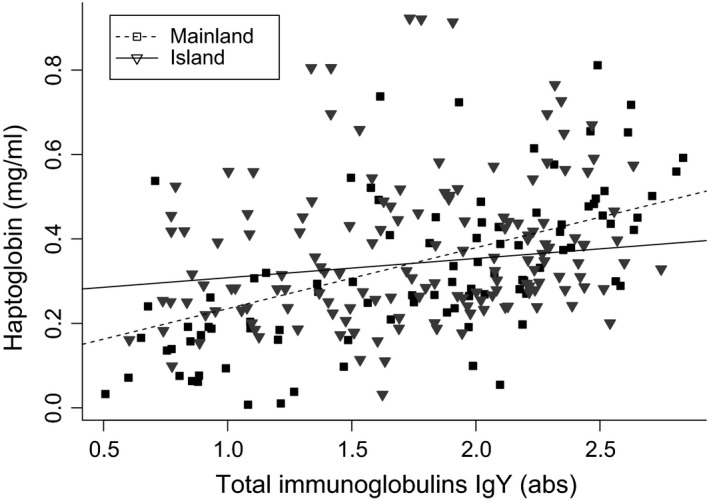
Positive association of total immunoglobulins and haptoglobin levels in birds from the Gulf of Guinea. Triangles and full lines:island birds; squares and dotted lines: mainland birds

In the subset of species excluding Estrildidae and Nectariniidae, both island and mainland species showed a positive association between haptoglobin and antibodies but mainland birds showed a sharpest slope (Table 3, Figure 3). Haptoglobin was positively associated with plasma redness (Table 3). The only significant interaction was between antibodies and insularity (χ^2^ = 8.04, *p* = .005). When analyzing the two subsets of pair composition separately (same species, different species), both subsets showed a positive association with plasma redness prescan absorbance (*p* < .001). In the subset including pairs with same species composition, haptoglobin was significantly and positively associated with body mass (*p* = .037) but the interaction antibodies and insularity was not significant (χ^2^ = 1.62, *p* = .20), while in the subset including pairs with different species, the interaction antibodies and insularity was significant (χ^2^ = 4.86, *p* = .027).

**Table 3:** Association of haptoglobin and total immunoglobulins in birds from the Gulf of Guinea. Estimates correspond to average slopes for continuous explanatory variables, average differences between levels of the factors, and average difference in slopes for a certain factor level in interaction term. Statistics of variables are shown in bold when *p*‐value <.05

	Value estimate	*SE*	*t*‐value	*p*‐value
Haptoglobin *N* = 247				
(Intercept)	−0.0074	0.058	−0.127	.90
Total immunoglobulins IgY	0.023	0.021	1.09	.28
Insularity (mainland)	**−0.13**	**0.050**	**−2.61**	**.010**
Plasma redness	**1.01**	**0.060**	**16.67**	**<.0001**
Body mass	−0.00039	0.00032	−1.21	.23
Sex (female)	−0.016	0.012	−1.30	.19
Status (nonendemic)	−0.089	0.039	−2.27	.057
Pair composition (same)	0.024	0.042	0.58	.62
IgY: Insularity (mainland)	**0.084**	**0.030**	**2.83**	**.0051**

## 4 Discussion

The aim of this study was to investigate whether there is a decrease in two immune parameters of island species or, alternatively, whether there is a differential investment between two components of the two different axes of immunity. Our study on the birds of the Gulf of Guinea found a reduction in total immunoglobulins on islands, whereas there was no significant difference in haptoglobin levels. We also observed a positive correlation between total immunoglobulins and haptoglobin levels indicating that there was no trade‐off between these two proxies of acquired and innate immunity, although this relationship appeared weaker on the islands. Our results provide an indication of a decrease in acquired but not in innate immunity parameters on the birds from these islands.

### 4.1 Different effects of insularity on antibody and haptoglobin levels

The reduction in antibody levels we documented here for the insular species is in agreement with the variation in antibody parameters found in previous studies. Specifically, in species from the Galapagos, there was a slower antibody response in ground finches (*Geospiza fuliginosa*; Lindström et al., 2004) and a less variable immune response in hawks (*Buteo galapagoensis)* on more inbred populations from smaller islands (Whiteman et al., 2006). Similarly, cell‐mediated immunity and leukocytes were found to be lower in the endemic Chatham Islands Forbes’ parakeet (*Cyanoramphus forbesi*) than in the cosmopolitan red‐crowned parakeet (*C. novaezelandiae;* Tompkins et al., 2006). However, not all the studies found such a reduction. A study comparing four avian taxa from remote Pacific islands with their closely related counterparts found no consistent reduction in natural antibodies in the island taxa (Beadell et al., 2007). A similar result was obtained in a study that compared 15 phylogenetically matched pairs of birds from mainland North America and island bird populations from Bermuda, Galápagos, and Hawaii, where natural antibodies did not change systematically among island–continent pairs (Matson, 2006).

Total antibody levels may incorporate the recent history of pathogen exposure and may also reflect investment in immunological function (Adamo, 2004; Apanius & Nisbet, 2006; Kelly, Murphy, Tarvin, & Burness, 2012; Morales et al., 2004). However, regardless of whether our results arise from reduced exposure to pathogens (either reduced prevalence of pathogens or reduced parasite diversity that translates in less total antibodies responding to different infections), or reduced investment in immunity, they are in agreement with a lower pathogen pressure on islands and thus with an “enemy‐release” effect (Keane & Crawley, 2002; MacArthur & Wilson, 1967; Torchin et al., 2001). As maintaining immune function is costly (Bonneaud et al., 2003; Lochmiller & Deerenberg, 2000), in the long term, lower pathogen pressure could lead to selection for a reduced investment in humoral responsiveness.

Contrary to expectations, and to the results obtained for the antibodies, we found no overall decrease in haptoglobin levels in the island birds sampled. Some pairs showed higher levels of haptoglobin on islands and others showed a reduction or no change, resulting in the absence of a significant global effect of insularity on haptoglobin concentration. The study conducted by Matson (2006) on 15 pairs of island–mainland bird species found an increase in haptoglobin levels (see also: Matson, Mauck, Lynn, & Tieleman, 2014), even though some other immune parameters decreased (see above; Matson, 2006; Matson et al., 2014). Based on those and our results, it appears that insularity does not lead to a reduction in levels of haptoglobin. This could suggest that its importance for survival may select for stability in haptoglobin values, which have been shown to remain constant through the annual cycle in birds (Hegemann et al., 2013).

It has been suggested that a reorganization of the immune system may take place in insular animals if factors associated with island life favor an adaptive shift to one type of defense strategy at the cost of other immune components (e.g., innate vs. acquired immunity; see (Lindström et al., 2004; Matson, 2006). Thus, inflammatory responses arising from infection or trauma could, to some extent, compensate the lower investment in acquired humoral response. If that was the case for the species studied here, we expected to find a negative correlation between haptoglobin levels (innate) and the total immunoglobulins Y levels (acquired). However, our data showed a positive correlation between both measures of immune function. This, however, does not preclude the possibility of trade‐offs between other immune components not measured here, and further study on this is required. We observed however a significant interaction showing a weaker association between both parameters in island species, a pattern mainly driven by pairs composed of nonconspecifics. This could indicate that longer isolation times may lead to changes in immunological strategies of mobilization of immune components.

### 4.2 Patterns in immune parameters and the island environment

The results obtained in this study for total antibodies and haptoglobin levels add to results from previous studies suggesting a nonuniform effect of insularity on immune parameters. Several factors may explain such variation in the response to insularity. First, in our study, the significant decrease in antibody levels was mainly driven by the same species paired comparisons suggesting that current conditions of the island environment can rapidly influence this immune parameter. In pairs composed of different species, the time since divergence may have allowed the immune system to follow independent evolutionary trajectories on the island and mainland species.

Second, fine‐scale differences in the level of host–parasite interactions, together with distinct characteristics of each island or within‐island environments, may lead to complex responses to insularity. For example, the observed relatively high antibody levels in three species from Príncipe Island (two endemics species *Anabathmis hartlaubii, Ploceus princeps* and the endemic subspecies *Aplopelia larvata principalis*) suggest that specific ecological conditions on particular locations can stand out over a global insularity effect (Apanius, Yorinks, Bermingham, & Ricklefs, 2000). Coincidentally, two of these endemic species have been shown to experience a high prevalence of blood parasites at the time the immunological parameters have been taken (*Anabathmis hartlaubii* and *Ploceus princeps,* (C. Loiseau, M. Melo, E. Lobato, J.S. Beadell, R.C. Fleischer, S. Reis, C. Doutrelant, R. Covas, unpublished data)). Third, it is also likely that species‐specific historical circumstances of island colonization may shape immune parameters. Even in the extremely isolated and parasite‐poor habitats such as in the Hawaiian Islands, at least one species has been shown to have a resistant genotype against introduced parasites (Foster et al., 2007).

Other mechanisms may contribute to explain the differences in immunological patterns observed between the insular and mainland species compared here. For example, life‐history changes in island animals, such as a shift toward a slower pace of life in island species (Covas, 2012; Novosolov et al., 2013), are thought to lead to a greater investment in individual quality, and this could counterbalance the tendency to reduced immune response on islands (Lee, 2006). In agreement with this hypothesis, a positive correlation of natural antibodies and longer developmental times has been observed in tropical birds (Lee, Wikelski, Robinson, Robinson, & Klasing, 2008). However, other studies found no reduction in antibodies and other immune parameters in species showing fast or slow reproductive pace when comparing mice (Martin, Weil, & Nelson, 2007) and bird species (Hasselquist, 2007; Horrocks et al., 2012). Further studies on life‐history changes on island species and how these correlate to immune parameters are needed to provide clarification on this hypothesis.

### 4.3 Conclusions and future studies

The simplicity of species‐poor island ecosystems, combined with emblematic cases of species extinction on islands following the introduction of exotic diseases, have led biologists to make predictions of islands animals as experiencing reduced disease pressure and, as a result, evolving weaker immunological systems when compared to their mainland counterparts. The present study adds to the few empirical studies conducted over the last 15 years that suggest a more complex picture.

Clearly, more studies are needed to clarify whether there is a general pattern of parallel evolution regarding the immune system of islands hosts. A broader survey of species and islands with different characteristics (i.e., islands with different sizes and degree of isolation in distinct climatic and geographic regions, as well as species from distinct phylogenetic groups) is needed. Moreover, the great complexity of the immune system needs to be further studied using more complete immune profiles and measurements made before and after immunological challenges, as well as specific immune responses to actual parasites that could be closely related to fitness (Nussey et al., 2014). Although these tests are challenging to conduct on a large number of species and locations, they are needed to better characterize immune systems (see also Matson, Cohen, Klasing, Ricklefs, & Scheuerlein, 2006). Finally, the host species’ life‐history characteristics should ideally be taken into account in future studies. Selective pressures to decrease investment in immune function on islands might be countered by a greater investment in self‐maintenance required in long‐lived species (Lee, 2006), but whether or not this mechanism takes place remains to be studied.

## Conflict of Interest

The authors declare they have no conflicts of interest.
